# Dye-Doped ZnO Microcapsules for High Throughput and Sensitive Optofluidic Micro-Thermometry

**DOI:** 10.3390/mi11010100

**Published:** 2020-01-17

**Authors:** Najla Ghifari, Sara Rassouk, Zain Hayat, Abdelhafed Taleb, Adil Chahboun, Abdel I. El Abed

**Affiliations:** 1 Laboratoire de Photonique Quantique et Moléculaire (LPQM), UMR 8537, Ecole Normale Supérieure Paris Saclay, CentraleSupélec, CNRS, Université Paris-Saclay, 94235 Cachan, France; 2 Laboratoire des Couches Minces et Nanomatériaux (CMN), FST Tanger, Université Abdelmalek Essaadi, 90000 Tangier, Morocco; adchahboun@uae.ac.ma; 3 PSL University, Chimie ParisTech—CNRS, Institut de Recherche de Chimie Paris, Paris 75005, France; Sorbonne université, 4 place Jussieu, 75231 Paris, France

**Keywords:** micro-thermometry, laser induced fluorescence, droplet microfluidics, zinc oxide, rhodamine B, rhodamine 6G

## Abstract

The main objective of this work is to show the proof of concept of a new optofluidic method for high throughput fluorescence-based thermometry, which enables the measure of temperature inside optofluidic microsystems at the millisecond (ms) time scale (high throughput). We used droplet microfluidics to produce highly monodisperse microspheres from dispersed zinc oxide (ZnO) nanocrystals and doped them with rhodamine B (RhB) or/and rhodamine 6G (Rh6G). The fluorescence intensities of these two dyes are known to depend linearly on temperature but in two opposite manner. Their mixture enables for the construction of reference probe whose fluorescence does not depend practically on temperature. The use of zinc oxide microparticles as temperature probes in microfluidic channels has two main advantages: (i) avoid the diffusion and the adsorption of the dyes inside the walls of the microfluidic channels and (ii) enhance dissipation of the heat generated by the focused incident laser beam thanks to the high thermal conductivity of this material. Our results show that the fluorescence intensity of RhB decreases linearly with increasing temperature at a rate of about −2.2%/°C, in a very good agreement with the literature. In contrast, we observed for the first time a nonlinear change of the fluorescence intensity of Rh6G in ZnO microparticles with a minimum intensity at a temperature equal to 40 °C. This behaviour is reproducible and was observed only with ZnO microparticles doped with Rh6G.

## 1. Introduction

With the advent of microfluidics and the development of many lab on a chip (LOC) applications, the need to ensure a good control and monitoring of temperature at the microscale with a high spatial and temporal accuracy started to merge. For instance, in biology and medicine, droplet microfluidics based on polymerase chain reaction, or digital polymerase chain reaction (dPCR), proved to be a highly sensitive technique for gene detection and gene sequencing [1,2]. This powerful technique relies on three major steps, which are performed at three different temperatures (95 °C, 54 °C and 72 °C) and repeated for 30 or 40 cycles to amplify a diluted template deoxyribonucleic acid (DNA) sample enabling to reach a detectable amount of suitable fluorogenic probes. Nevertheless, despite some progress [3], the realization of an integrated microfluidic digital PCR microchip, where the whole PCR thermo-cycling process can be realized, is still hampered by the difficulty to measure and to control precisely the temperature inside the microfluidic channels.

During the last two decades, several methods, mainly based on micro-electro-mechanical systems (MEMS) technology, have been developed to ensure a good thermal control within micro-systems and micro-devices [4]. Nevertheless, most of these methods suffer from several limitations such as high cost, complicated manufacturing and poor spatial resolution. Laser induced fluorescence (LIF) technique has proven to circumvent many of these limitations [3,4,5,6,7,8,9,10,11,12,13,14]. Indeed, many fluorescent dyes exhibit a fluorescence intensity which depends strongly on the temperature. Therefore, such dyes may be used as molecular probes to measure temperature in a highly sensitive and very localized manner, both in space and time. Among the most efficient molecular probes for temperature measurements, rhodamine B (RhB) is the most widely used.

At low concentrations, the fluorescence intensity $I_{f}$ (per volume unit) of a fluorescent dye depends generally on several parameters according to the following linear relation, which may be derived simply from Beer-Lambert law [15,16,17]:

(1) $$I_{f}\propto \Phi I_{0}\epsilon C$$

where Φ, $I_{0}$, ϵ and *C* are the fluorescence quantum yield, incident illumination intensity, molar absorptivity and dye concentration, respectively. For most temperature sensitive dyes, likewise RhB, it is generally observed that an increase in temperature results in a decrease of the fluorescence quantum yield Φ and the fluorescence life time. This is because the non-radiative processes related to thermal agitation (collisions with solvent molecules, intra-molecular vibrations and rotations) are more efficient at high temperatures [15,18]. One may cite nevertheless, the particular cases of rhodamine 6G (Rh6G) whose fluorescence increases linearly with temperature [6] and rhodamine 110 (Rh110) whose fluorescence does not depend on temperature. For the latter compounds, the increase or stability of fluorescence intensity versus temperature may be attributed to the formation of complexes (dimers, trimers,…). Such complexes are better shielded from inter-molecular collisions and have higher quantum yields leading hence to an increase or a stability of fluorescence intensity versus temperature [18]. As can be seen from the above equation (Equation (1)), factors such as a non-uniform illumination ($I_{0}$) or a non-uniform dye concentration *C* can lead to major errors and uncertainties on temperature measurement.

While uncertainty caused by a fluctuation of the laser illumination can be eliminated relatively easily by normalizing fluorescence intensity using a reference temperature-insensitive dye, such as Rh110, those which are caused by a fluctuation of the dye concentration in the microfluidic device are much more difficult to handle. Indeed, several processes can be at the origin of concentration fluctuations, such as adsorption of dye molecules on the channel walls [7], trapping of molecules inside the porous substrate of the microfluidic device (e.g., PDMS) or a non homogeneous distribution of the flow advected dye molecules along the microchannel, caused by the well-known Taylor dispersion phenomena [19,20]. The existence of all these processes may result in a local depletion or enrichment of the dye in the microfluidic device.

One should emphasize also another potential and important source of uncertainty on the measure of temperature in microfluidic systems, that is the heat dissipation within a tiny volume illuminated by a focused laser beam. To our knowledge, such an effect has been often neglected in the literature. In order to minimize the increase of temperature caused by the heating of the incident laser beam, one should either use a highly sensitive detection setup enabling to minimize the illumination power and/or increase the efficiency of the heat dissipation in the illuminated area of the sample by using a suitable material with a high thermal conductivity.

**Figure 1:**
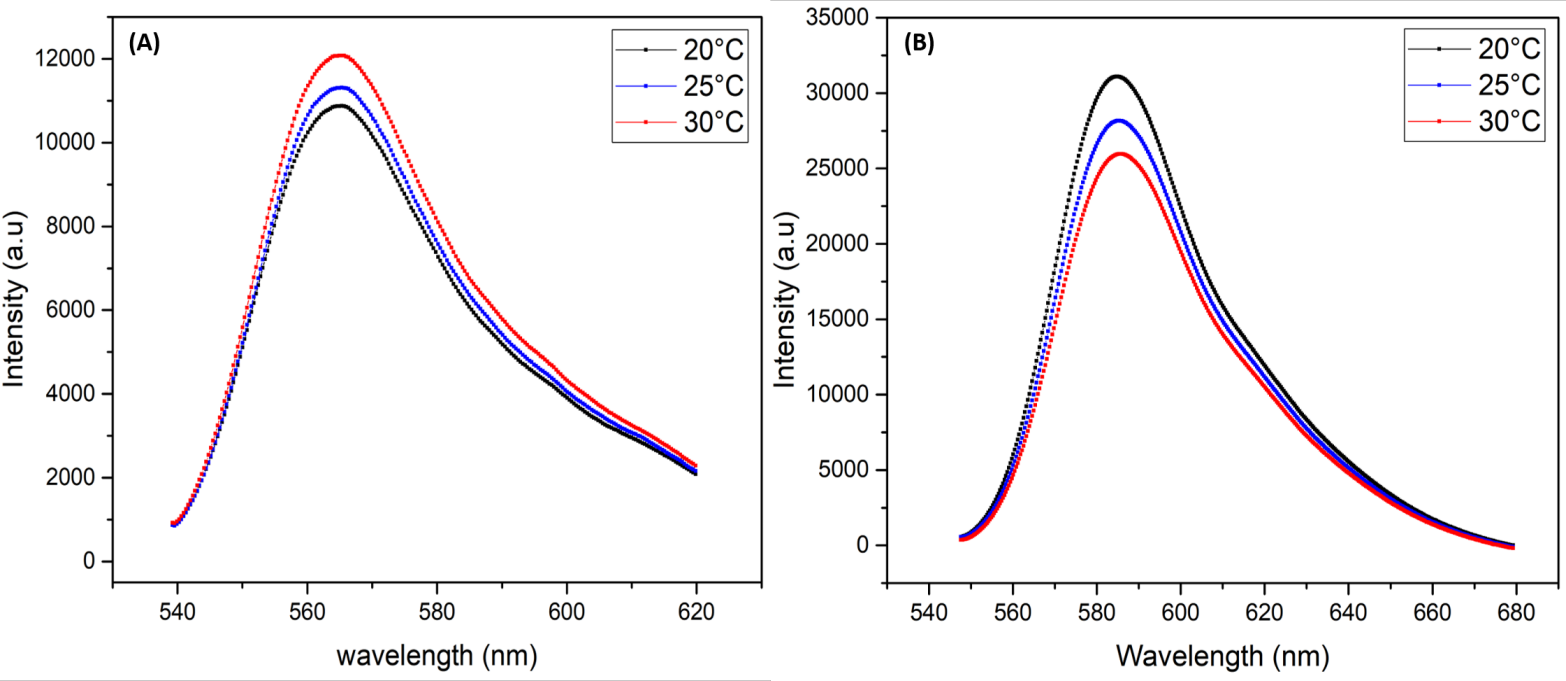
Fluorescence spectra of Rhodamine 6G (**A**) and Rhodamine B (**B**) versus temperature.

The main objective of this work is to give a proof of concept of a new approach for measuring the temperature inside microfluidic channels at high throughput and in a reliable manner using laser induced fluorescence (LIF) technique of rhodamine B dye confined in highly monodisperse zinc oxide microspheres. Indeed, besides its high chemical stability and photo-stability, zinc oxide exhibits a high thermal conductivity of about 100 to 150 W/m·K (depending on ZnO structural properties) [21,22,23]. This value is for instance two orders of magnitude higher than the thermal conductivity of water, i.e., 0.65 W/m·K. We used three types of dye doped ZnO microparticles: one type is doped with RhB, a second type is doped with Rh6G and the third type is doped with a mixture of RhB and Rh6G. The later mixture is intended to be used as a reference for fluorescence intensity calibration and to take account of fluctuations in incident illumination intensity. Indeed, the fluorescence intensity of RhB decreases linearly with increasing temperature at a ratio of about −2%/°C whereas the fluorescence intensity of rhodamine 6G (Rh6G) increases linearly with increasing temperature at a rate of about +2%/°C [6,12,24,25], as shown (see Figure 1).

## 2. Experimental Section

### 2.1. Synthesis of ZnO Nanoparticles Building Units

We first synthesized ZnO nanoparticles which serve as building blocks for the final ZnO microparticles using sol-gel technique. Then, we confined such nanoparticles in highly monodisperse droplets using a flow-focusing microfluidic device in order to fabricate highly monodisperse ZnO microspheres where RhB and Rh6G are confined in a controlled manner.

The synthesis of colloidal ZnO nanoparticles can be carried out using different types of precursors dissolved in alcohol solvents [26]. We used zinc acetate dehydrate Zn(CH${}_{3}$COO)${}_{2}$:2H${}_{2}$O (99.999%, from Sigma-Aldrich France, St. Louis, MO, USA) as ZnO precursor and methanol as a solvent, following the procedure which is described in more detail in [27]. All reagents were of analytical grade and were used as received without any further purification. Briefly, we dissolved 0.6 g in 5 mL of methanol (CH${}_{3}$OH), then, the solution was stirred during 1 h at 60 °C under magnetic stirring to ensure homogeneous mixing and obtain a transparent solution.

The mechanism of formation of zinc oxide nanoparticles used in this work is according to a succession of chemical reactions. First, the dissolution of dehydrated zinc acetate in the presence of methanol contributes to the dehydration of zinc oxide precursor which results in the formation of anhydrous zinc acetate and water. Followed by a preliminary dissolution of the anhydrous zinc acetate to form the zinc ion and acetate. The latter will become acetic acid. It then takes place a chemical reaction between the species present (zinc and hydroxide ions) in the solution causing the precipitation of zinc hydroxide, which is ultimately converted to ZnO nanoparticles. Finally, the resulting dispersion of ZnO nanoparticles (with residual solvents) is doped with the desired fluorescent dye/dyes. Subsequently, the resulting mixture was immediately injected into the microfluidic chip to generate highly monodisperse microdroplets. The formation mechanism of zinc oxide nanoparticles can be summarized in these chemical reactions:

$${\mathrm{Zn}(\mathrm{CH}}_{3}{\mathrm{COO})}_{2}\cdot 2H_{2}O⟶{\mathrm{Zn}(\mathrm{CH}}_{3}{\mathrm{COO})}_{2}+2H_{2}O$$

$${\mathrm{Zn}(\mathrm{CH}}_{3}{\mathrm{COO})}^{2-}⟶{\mathrm{Zn}}^{2+}+2{\mathrm{CH}}_{3}{\mathrm{COO}}^{-}$$

$${\mathrm{Zn}}^{2+}+2{\mathrm{OH}}^{-}⟶{\mathrm{Zn}(\mathrm{OH})}_{2}$$

$${\mathrm{Zn}(\mathrm{OH})}_{2}⟶\mathrm{ZnO}+H_{2}O$$

### 2.2. Fabrication of Microfluidic Devices and Synthesis of ZnO Microparticles

**Figure 2:**
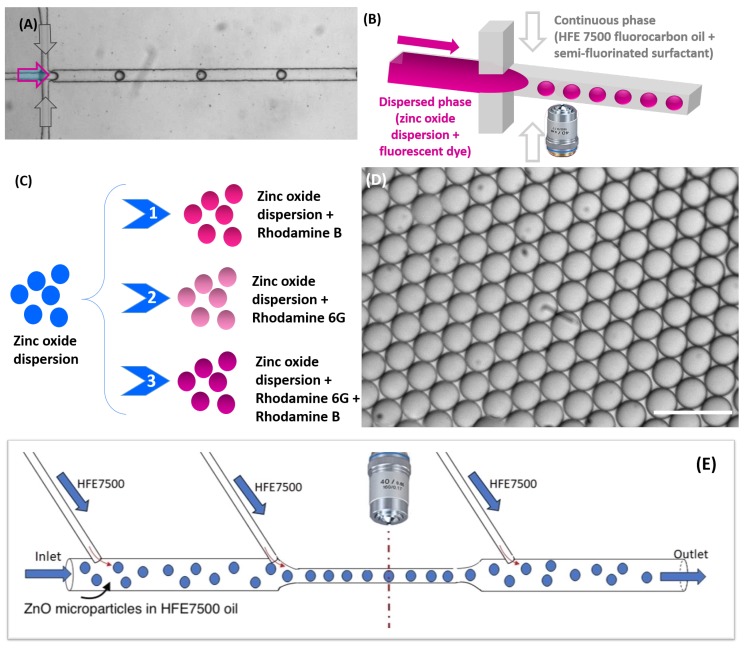
(**A**) Optical micrograph and schematic illustration of (**B**) the flow-focusing microfluidic geometry for zinc oxide droplet formation; (**C**) and the different case studies; (**D**) Optical micrograph of stable and monodisperse zinc oxide droplets generated through the flow-focusing microfluidic device, Scale bar, 100 μm; (**E**) Schematic illustration of the used microfluidic design for fluorescence analysis of doped ZnO microparticles.

Microfluidic devices were manufactured according to the conventional soft lithography technique [28]. In a first step, a pattern was transferred to SU-8 photoresist, previously coated on a silicon wafer, followed by the exposure to ultraviolet (UV) light, through the mask pattern. UV illumination leads to the polymerization of the photoresist located under the transparent regions of the mask. After development, the master mold is ready for the next step. In a second step, the polydimethylsiloxane (PDMS) was first mixed with a cross-linking agent with a weight ratio of 10:1, the mixture then was degassed using a vacuum pump at room temperature and the solution was poured onto the previously fabricated mold and placed in the oven for polymerization at 75 °C for 2 h. The block of PDMS was then removed from the mold; we thus obtain a replica of microchannels. In a third step, the PDMS block and the glass slide were treated with oxygen plasma for 20 s to enable their bonding and sealing the microfluidic chip. The design of the microfluidic device for droplets generation contained two inputs, one input for the carrier oil and a second input for the dispersed phase, a main (square) channel cross section of about 80 μm × 80 μm and an output for the collection of droplets in a Petri dish (see Figure 2a,b). The flow rates of the carrier oil ($Q_{c}$) and the dispersed phase (ZnO dispersion) ($Q_{d}$) were set using Nemesys syringe pumps (Cetoni GmbH, Korbussen, Germany). The dispersed phase consisted of ZnO solution doped with one of the selected rhodamine dyes: RhB, Rh6G and a mixture of both dyes. The flow rates were set in order to produce droplets with the same size (55 μm) for all experiments. Then, the droplets were collected in Petri-dish with HFE 7500 at room temperature.

The used carrier oil phase consisted of a fluorocarbon oil (HFE 7500, 3-ethoxy-dodecafluoro-2- trifluoromethyl-hexane, Inventec, Bry-sur-Marne, France), with a density of 1.61 g/cm${}^{3}$ and thermal diffusivity of about $\kappa =3.6\times 10^{-8}$ m${}^{2}$/s; 0.2 % (*w*/*w*) of a commercial surfactant (dSURF, Fluigent, Le Kremlin-Bicêtre, France) was added in HFE 7500 oil in order to prevent droplets merging. The used fluorocarbon oil has the advantage of not inducing PDMS swelling and being also chemically inert. More important for our application, it does not solubilize any of the ZnO precursor solution components.

After their generation, droplets were transported along the microfluidic channel by the carrier oil phase (see Figure 2) and then collected in a Petri dish (not shown on Figure 2), where droplets formed a floating monolayer at the oil-air interface, because of the higher density of fluorocarbon oil ($d_{HFE-7500}=1.62$). In our study, the condensation of ZnO droplets was mainly controlled by the evaporation of the solvent at the oil–air interface. We let the condensation performing until microparticles reach their final sizes: 17.8 ± 0.2 μm for RhB doped microparticles and 20 ± 0.2 μm for both Rh6G and RhB/Rh6G doped microparticles.

For the re-injection of the synthesized microparticles, we used another microfluidic device (re-injector), which was made of a main inlet enabling the injection of the dispersed ZnO microparticles in HFE 7500 oil and three other secondary inlets for the injection of pure carrier oil to prevent aggregation of microparticles and the clogging of the microfluidic channel. The design of the re-injector includes also a constriction of the rectangular channel, with a width *w* = 22 μm and a height *h* = 38 μm. The overall flow rate in this constriction area was about *Q* = 150 μL/h. Therefore a mean flow velocity of about $v_{constr.}=\frac{Q}{w\times h}≃5$ cm/s can be deduced.

### 2.3. Fluorescence Detection of Flowing Microparticles

**Figure 3:**
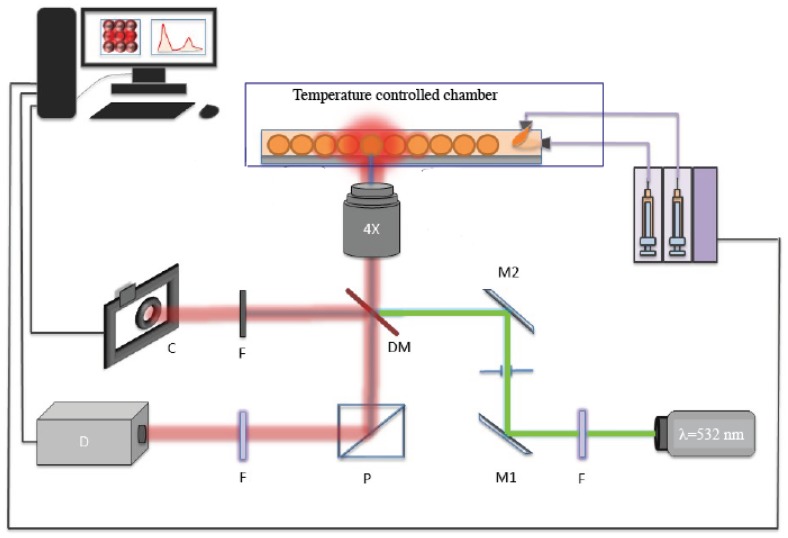
Experimental setup for high throughput fluorescence measurements.

Figure 3 shows the home-built fluorescence acquisition setup used in this study. It enables for a highly sensitive fluorescence detection, which is described in detail in Ref. [29]. It includes multiple laser sources optimised for the absorption of different fluorophores (we used in this study one continuous-wave (CW) source with wavelength of 532 nm). Laser incident beams are combined by mean of a first dichroic mirror (DM1) and then directed towards microdroplets in the microfluidic channel by mean of a second dichroic mirror (DM2). The focused band limited light is targeted towards the droplets/particles and recollected by the microscope objective, which is then transmitted through another set of band-limited filters to two photo-multiplier tubes (PMT’s). The signal outputs from PMT’s are then collected at high acquisition rates (100 KHz) using a DAQ acquisition card (National Instruments) and analyzed them using LabVIEW and a FPGA (field programmable gate arrays) module scripts, which allows for the identification of droplets by the modulation of fluorescence versus time. Depending on the selected sampling acquisition of the fluorescence signal, the width of the time lapse for signal detection can be varied from 40 ms (max sample rate of 200 KHz) to seconds or minutes.

### 2.4. Calibration of Temperature Measurements

We used a thermo-plate heating chamber (Tokai-Hit, Shizuoka, Japan) to control precisely the temperature of the whole microfluidic device (±0.1 °C). This device is equipped with two heating plates, one at the bottom and one at the top. The later consists of a large clear glass top heater providing a uniform temperature distribution in the whole chamber. The heating device is also equipped with a feedback sensor mechanism that enables a real-time, precise sample temperature feedback temperature regulation. The heating chamber was placed on IX73 Olympus microscope (OLYMPUS, Tokyo, Japan) stage to measure the fluorescence of ZnO particles doped with rhodamine at different temperatures from 20 to 50 °C.

To determine the dependence of the fluorescence intensity versus temperature, we enclosed the entire microfluidic chip in the heating chamber. For each temperature value, we waited at least 10 min before recording the fluorescence intensity. Also, in order to give enough time for the incoming microparticles and HFE 7500 oil to reach the selected (target) temperature, a length of the tubing inlets (ZnO microparticles dispersion and pure fluorocarbon oil) of about L≃10 cm was enclosed in the heating chamber. Taking account of the used overall flow rate, Q≃150 μL/h and the inner diameter of the tubing, $D_{tub.}=0.56$ mm, one may deduce a flow velocity in the tubing of about $v_{flow}=\frac{Q}{\pi {\left(D_{tub.}/2\right)}^{2}}≃4$ cm/min.

This means that it should take less than 3 min for the incoming fluid and microparticles to flow from the entry of the heating system until the inlet of the microfluidic channel, where fluorescence intensity is measured.

Considering now the thermal diffusivity of HFE 7500 oil, that is 2.16 mm${}^{2}$/min, and the cross section size of the tubing, one may calculate a diffusion time as small as $t_{diff}≃4$ s, which is the time needed for the temperature to diffuse and become homogenized through all the cross section of the flowing fluid. As one may notice, the calculated diffusion time is much shorter than the flow time of the fluid before it reaches the area of the microfluidic channel where the temperature is measured.

## 3. Results and Discussion

### 3.1. Fluorescence Intensity Versus Temperature of ZnO Microparticles Doped with rhodamine B (RhB)

**Figure 4:**
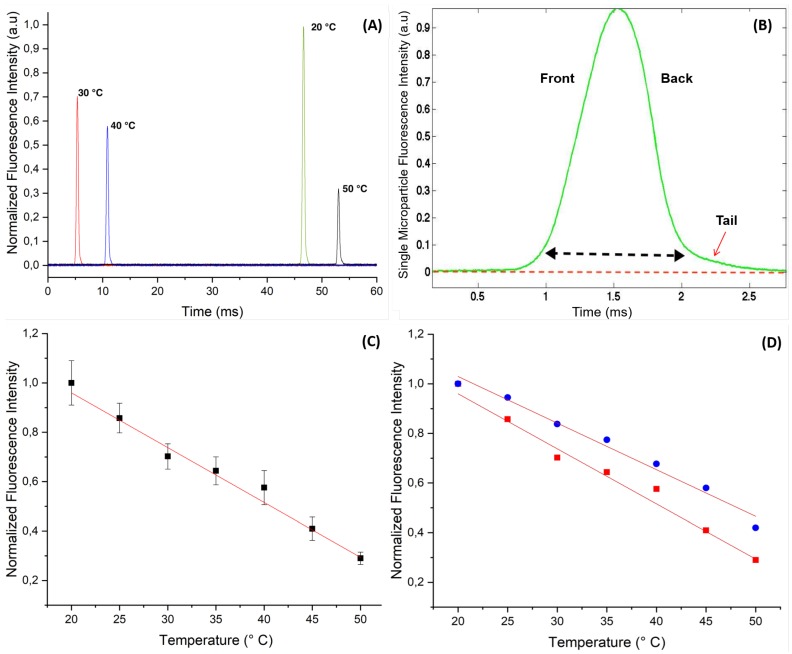
(**A**) Recorded fluorescence intensity peaks at different temperature from confined rhodamine B dye in flowing ZnO microcapsules (with a size of 17.8 μm) along a 22 μm wide micro-channel constriction area; (**B**) High-magnification of the fluorescence intensity peak vs. time of doped ZnO microcapsules with rhodamine B; (**C**) Plot of the linear decrease of the normalized fluorescence intensity of doped ZnO microparticles with rhodamine B vs. temperature; (**D**) Comparison of the change of fluorescence of RhB vs. temperature in flowing RhB doped ZnO microparticles (squares, red curve) and flowing RhB doped ZnO microdroplets (circles, blue curve), the slope of the two curves are very close and in very good agreement with the literature data: (−2.2±0.1)%/°C and (−1.9±0.2)%/°C, respectively.

Figure 4 shows the change versus different temperatures, ranging from 20 to 50 °C, of the fluorescence intensity of RhB doped ZnO microparticles carried by a flow of HFE 7500 of about 150 μL/h in the microfluidic channel.

We first show in Figure 4A the fluorescence intensity peaks of individual flowing microparticles as they were detected and recorded from different experiments (at different temperatures). Because microparticles were randomly dispersed in the carrier oil, the time at which microparticles crossed the constriction area of the microfluidic channel, where they were detected, was random and hence the origin of the time axis was arbitrary. Nevertheless, one could have the measured value of the width of each peak to roughly estimate the size of the microspheres, which was found here to be about 1.5 ms, as shown in Figure 4B.

Taking account the mean flow velocity of 5 cm/s in the constriction area of the microchannel, one deduces a microparticle size of about L≃75 μm, which was four times the size we measured from microscopy image analysis, $D_{RhB}=17.8\pm 0.2$ μm. Noteworthy, we made the assumption that microparticles flow with the same mean velocity as the surrounding carrier fluid’s, which was basically not a good approximation, because of the presence of a thin lubrication film of the continuous phase between the particles and the channel walls as reported in the literature [30,31].

It is also interesting to notice from Figure 4B that the fluorescence peaks exhibited an asymmetric bell shape with an elongated tail at the back side of the microparticle. This observation, with other results obtained from optical and scanning electronic microscopies (not shown in this study), corroborates the relative softness of the synthesized microparticle and their deformability. We have shown in a previous study that the synthesized microparticles consist of microcapsules with a hollow structure and a thin envelope whose thickness value was found to be about 0.7 μm [27]. The group of Slasac and Barhès-Biesel et al. have shown for instance that microcapsules can deform easily under the effect of a shear flow in a microchannel [32,33].

One of the main advantage of droplet microfluidics based synthesis approach is the ability to obtain highly monodisperse microparticles, which in turn enabled us to obtain results with very good statistics. We normalized the fluorescence intensity values by considering the intensity recorded at 20 °C as a reference, $I_{20{}^{\circ}C}=1$.

For each temperature, we collected the fluorescence signal from approximately 20 highly monodisperse microparticles and plot the average values with their standard deviation versus temperature, as shown in Figure 4C. This figure shows clearly that the fluorescence intensity of RhB doped ZnO microparticles decreased linearly vs. temperature with a rate of about (−2.2±0.1)%/°C. The determined slope was in very good agreement with the value reported generally in the literature for RhB dye [4,5,6,12,13,14].

In order to compare the effect of ZnO on the fluorescence behavior of RhB versus temperature, we present in Figure 4D the (normalized) fluorescence intensity of RhB when confined in microdroplets made of a water based RhB solution (0.5 mM and droplets size about 50 μm). We found that the slope of the linear decrease of the fluorescence intensity versus temperature was very close to the one recorded from flowing RhB doped ZnO microparticles, (−1.9±0.2)%/°C.

### 3.2. Fluorescence Intensity Versus Temperature of ZnO Microparticles Doped with rhodamine 6G (Rh6G)

**Figure 5:**
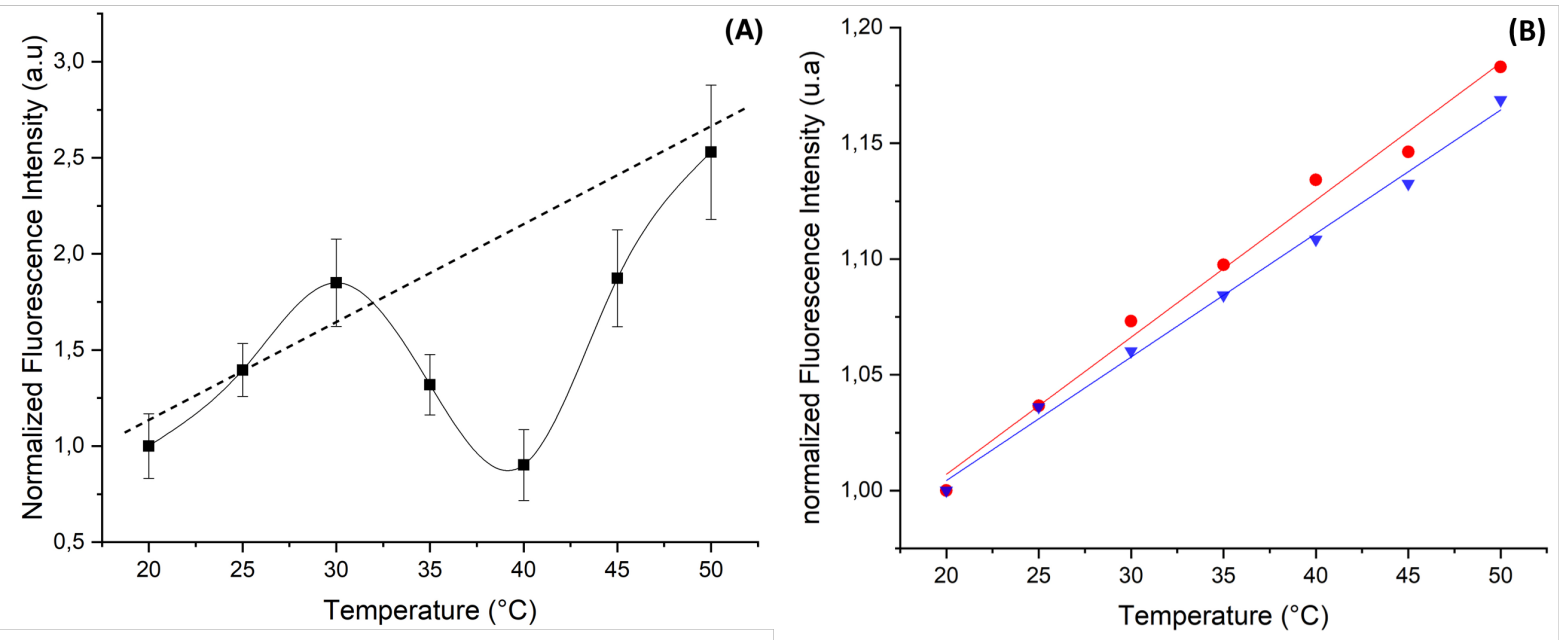
Change of the relative intensity of the fluorescence of rhodamine 6G (Rh6G) vs. temperature in (**A**) ZnO microparticles and (**B**) in water droplet (blue curve) and in ethanol droplet (red curve).

Figure 5A shows the change versus temperature of the fluorescence intensity of ZnO microparticles when doped with Rh6G dye. As may be noticed, though the fluorescence intensity appeared to increase globally with temperature, as observed in the bulk solution [6], we observed for the first time that the fluorescence intensity exhibited a minimum around 40 °C. This behavior is reproducible and was observed only when ZnO microparticles were doped with Rh6G.

Figure 5B shows a “standard” linear increase versus temperature of the of the fluorescence intensity of Rh6G when dissolved in microdroplets made of a 0.5 mM aqueous solution (droplet size equal to 50 μm) and when dissolved in a bulk solution. Nevertheless, the obtained slope for both curves, around 0.5%/°C, was much smaller than the one reported in the literature, 1.9%/°C [6]. Nevertheless, no minimum of intensity was observed in these cases, which indicates that this phenomena must be related to the confinement of Rh6G in ZnO microparticles.

**Figure 6:**
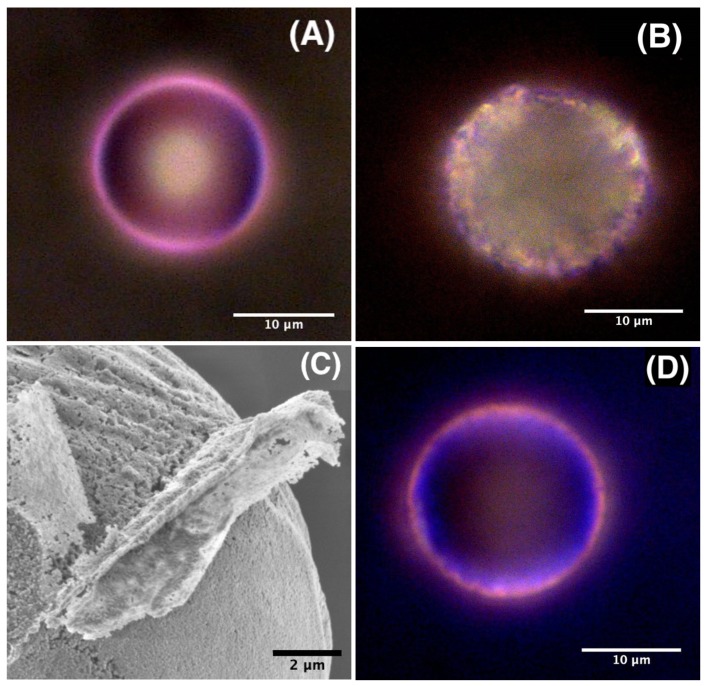
Optical microscopy images of doped ZnO microspheres with RhB (**A**), Rh6G (**B**) and a RhB/Rh6G mixture (**D**); (**C**) High resolution scanning electron microscopy image of a ZnO microparticle showing the aggregation of ZnO nanocrystal building units of the microsphere and the resulting porosity of the material.

Though, the aim of this study is to give a simple demonstration of the proof of concept of our dye doped ZnO microparticles approach for locally measuring the temperature, we present preliminary results obtained by optical and scanning electron microscopies, which enables to understand (partly) the observed difference between RhB and Rh6G dyes in ZnO microparticles, as shown in Figure 6.

Figure 6A shows that RhB molecules organized in a homogeneously distributed pink colored shell around ZnO microsphere, which corresponded basically, as stated earlier, to a microcapsule with a thin porous envelope having a thickness of about 0.7 μm, as shown in more detail in reference [27]. We emphasize in this study the porosity of the microcapsule shell, which resulted from the aggregation of ZnO nanoparticles building units, whose average size was found to be about 100 nm, and the formation of voids between such nanoparticles, as can be seen from the scanning electron microscope (SEM) image shown on Figure 6C. One may suggest also that these pores may serve as niches for aggregates of dyes molecules.

Also, since rhodamine molecules were added at the early stage of the formation of the microcapsules, i.e., in the microdroplet state, the observed colored ring on the surface of ZnO microsphere should correspond to an adsorbed layer of RhB molecules on the inner surface of the microcapsule. In contrast, Figure 6B shows that Rh6G dye molecules were less localized on the surface of the microcapsule and the dye seemed to occupy a diffuse and extended area all over the surface of the shell. Moreover, we observe the presence of many bright spots with different brightness, which revealed a non homogeneous distribution of Rh6G dye molecules inside the shell of the microcapsule. We attribute such brighter regions to pores and niches in the ZnO microcapsule shell where the dye accumulates more or less strongly. Moreover, though we have used the same size for the initial droplets (55 μm) and the same ZnO precursor concentration (0.25 mM) for both types of microcapsules, we found that those doped with Rh6G had a lager size (20 ± 0.2 μm) than those doped with RhB (17.8 ± 0.2 μm).

**Figure 7:**
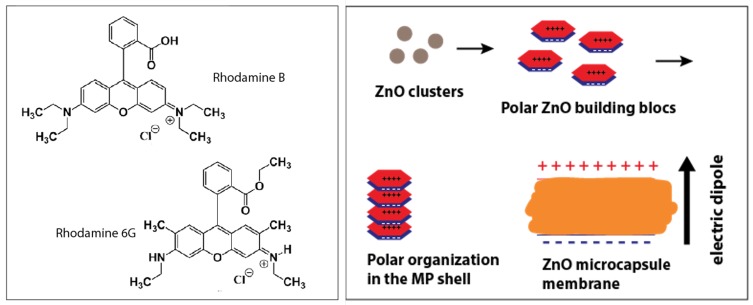
(**left**) Chemical structure of the used RhB and Rh6G dyes, depending on the pH, RhB molecules may exhibit either a zwitterionic form (presence of both a positive and negative charges are present) or may exhibit only a negative charge, whereas Rh6G molecules can carry only a positive charge or no charge at all, (**right**) illustration of the formation of the polar ZnO nanocrystals building units.

We suggest that the two different behaviors of the two dyes with ZnO microcapsules should be related to the electrical charges carried by the dyes and the electrically charged ZnO nanocrystals building blocks. Indeed, whereas RhB molecules can adopt (depending on the pH value) either a positively charged form or a zwitterionic form, for which the overall electrical charge is zero, Rh6G molecules can carry only a positive electric charge, as shown from their chemical structures in Figure 7A. Also, ZnO nanocrystals are known to carry a net electric dipole moment, which is inherent to the tetrahedral configuration of the wurtzite crystalline structure. In a such structure, each type of ion, Zn${}^{2+}$ or O${}^{2-}$, has four neighbouring ions of the other type of atom, and vice versa. That is to say, the tetrahedral coordination exhibits a sequence of positively charged Zn${}^{2+}$ and negatively charged O${}^{2-}$ polar planes within the c-axis direction, thus respectively contributing to two opposite faces of polarity (0001) and $\left(000\bar{1}\right)$ perpendicular to the c-axis. This results in an intrinsic electric dipole moment and a spontaneous polarization along the c-axis of the nanocrystals [34,35,36,37,38].

Therefore, one may expect that a strong interaction should occur between the positively charged Rh6G molecules and the negatively charged plane of ZnO nanocrystals, leading probably to a larger diffusion of Rh6G inside the porous structure of the microcapsule shell than could occur with RhB molecules in their zwitterionic form. At this stage, we still need further investigations in order to make a correlation between the adsorption of Rh6G dye in the pores of the shell and the observation of a minimum fluorescence intensity at a temperature around 40 °C.

### 3.3. Fluorescence Properties of ZnO Microparticles Doped with a Mixture of RhB and Rh6G

**Figure 8:**
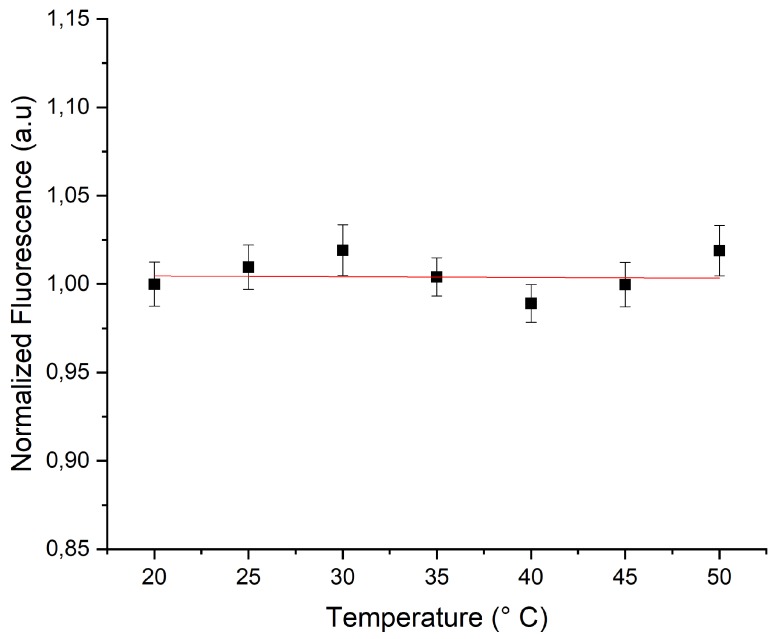
Relative intensity of the fluorescence of RhB and Rh6G mixture versus temperature in ZnO microparticles.

Figure 8 shows the fluorescence intensity versus temperature of ZnO microparticles which were doped with a mixture of RhB (29 μm) and Rh6G (37 μm) dyes. Because of the non linear variation of the fluorescence intensity of Rh6G in ZnO microparticles, it was difficult to predict the concentration ratio of the two dyes which would lead to a constant fluorescence intensity versus temperature. Nevertheless, after several attempts, we found that the ratio $\frac{\left[RhB\right]}{\left[Rh6G\right]}=\frac{29\;\mu M}{37\;\mu M}$ gave a good reference for which the fluorescence intensity remained practically constant versus temperature, as shown in Figure 8.

## 4. Conclusions and Perspectives

**Figure 9:**
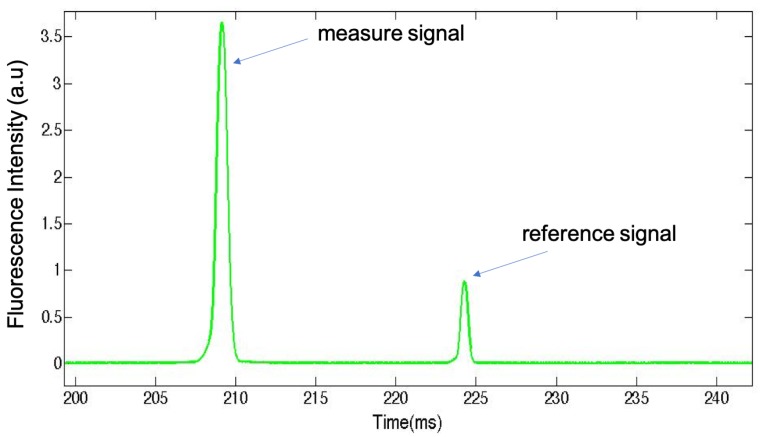
Different microparticles doped with two different dyes may be discriminated by the value of their fluorescence intensity signals. Such concentration based coding method will be applied in the future for the simultaneous recording of the fluorescence intensity signals from RhB doped microparticles (measure probes) and Rh110 doped microparticles (reference probes).

We have shown in the present study that ZnO microparticles doped with RhB dye exhibit a fluorescence intensity which decreases linearly versus temperature with a rate of about (−2.2±0.1)%/°C. This value is in very good agreement with the literature date. Our results should pave the way for the development of a promising technique which will enable in the future for a real time and highly sensitive measurement of the temperature in microfluidic channels. The use of ZnO microparticles for confining the temperature sensitive dyes has two main advantages: (i) it enables to circumvent the diffusion and the adsorption of the dyes on the walls of the microfluidic channel and inside the pores of the PDMS substrate of the microfluidic device, (ii) it enhances the dissipation of the heat generated by the focused incident laser beam thanks to the high thermal conductivity of ZnO material. In contrast with the fluorescence intensity linear change versus temperature of Rh6G in bulk solution or in microdroplets, Rh6G when confined in ZnO microparticles shows a non linear behavior of the fluorescence intensity versus temperature. The presence of a minimum at a temperature around 40 °C is reported for the first time. Such non linear behavior makes the construction of reliable reference a little bit cumbersome by nonetheless we determined experimentally a suitable mixture of RhB and Rh6G for which the fluorescence intensity remains practically constant in the investigated temperature range [20 °C–50 °C]. We envisage to consider in a future study the use of rhodamine 110 for the construction of such reference. Also, we plan to flow and detect simultaneously the two types of microparticles (RhB as a measure probe and Rh110 as reference probe) in the microchannel in order to make the normalization of the fluorescence intensity signal straightforward. For this purpose, the use of two different concentrations for the two types of microparticles will allow for the discrimination between the two types of particles while flowing at high throughput in the microchannel, as illustrated in Figure 9.

## References

[1] Pohl G., Shih I.M. (2004). Principle and applications of digital PCR. Expert Rev. Mol. Diagn..

[2] Taly V., Pekin D., El Abed A., Laurent-Puig P. (2012). Detecting biomarkers with microdroplet technology. Trends Mol. Med..

[3] Guijt R.M., Dodge A., van Dedem G.W., de Rooij N.F., Verpoorte E. (2003). Chemical and physical processes for integrated temperature control in microfluidic devices. Lab Chip.

[4] Gosse C., Bergaud C., Löw P. (2009). Molecular probes for thermometry in microfluidic devices. Thermal Nanosystems and Nanomaterials.

[5] Ross D., Gaitan M., Locascio L.E. (2001). Temperature measurement in microfluidic systems using a temperature-dependent fluorescent dye. Anal. Chem..

[6] Kuzkova N., Popenko O., Yakunov A. (2014). Application of temperature-dependent fluorescent dyes to the measurement of millimeter wave absorption in water applied to biomedical experiments. J. Biomed. Imaging.

[7] Samy R., Glawdel T., Ren C.L. (2008). Method for microfluidic whole-chip temperature measurement using thin-film poly (dimethylsiloxane)/Rhodamine B. Anal. Chem..

[8] Kim M.M., Giry A., Mastiani M., Rodrigues G.O., Reis A., Mandin P. (2015). Microscale thermometry: A review. Microelectron. Eng..

[9] Christofferson J., Maize K., Ezzahri Y., Shabani J., Wang X., Shakouri A. (2008). Microscale and nanoscale thermal characterization techniques. J. Electron. Packag..

[10] Erickson D., Sinton D., Li D. (2003). Joule heating and heat transfer in poly (dimethylsiloxane) microfluidic systems. Lab Chip.

[11] Arata H.F., Löw P., Ishizuka K., Bergaud C., Kim B., Noji H., Fujita H. (2006). Temperature distribution measurement on microfabricated thermodevice for single biomolecular observation using fluorescent dye. Sens. Actuators B: Chem..

[12] Liu G., Lu H. (2015). Laser-induced fluorescence of rhodamine B in ethylene glycol solution. Procedia Eng..

[13] Ebert S., Travis K., Lincoln B., Guck J. (2007). Fluorescence ratio thermometry in a microfluidic dual-beam laser trap. Opt. Express.

[14] Sakakibara J., Adrian R.J. (1999). Whole field measurement of temperature in water using two-color laser induced fluorescence. Exp. Fluids.

[15] Valeur B., Berberan-Santos M. (2013). Molecular Fluorescence: Principles and Applications. J. Biomed. Opt..

[16] Guilbault G.G. (1973). Practical Fluorescence, Theory, Methods and Techniques.

[17] Chamarthy P., Garimella S.V., Wereley S.T. (2010). Measurement of the temperature non-uniformity in a microchannel heat sink using microscale laser-induced fluorescence. Int. J. Heat Mass Transf..

[18] Hossain M.A., Canning J., Yu Z., Ast S., Rutledge P.J., Wong J.K.H., Jamalipour A., Crossley M.J. (2017). Time-resolved and temperature tuneable measurements of fluorescent intensity using a smartphone fluorimeter. Analyst.

[19] Taylor G. (1953). Dispersion of a solute in a solvent under laminar conditions. Proc. R. Soc. London, Ser. A.

[20] Beard D.A. (2001). Taylor dispersion of a solute in a microfluidic channel. J. Appl. Phys..

[21] Özgür Ü., Gu X., Chevtchenko S., Spradlin J., Cho S.J., Morkoç H., Pollak F., Everitt H., Nemeth B., Nause J. (2006). Thermal conductivity of bulk ZnO after different thermal treatments. J. Electron. Mater..

[22] Florescu D.I., Mourokh L., Pollak F.H., Look D.C., Cantwell G., Li X. (2002). High spatial resolution thermal conductivity of bulk ZnO (0001). J. Appl. Phys..

[23] Alvarez-Quintana J., Martínez E., Pérez-Tijerina E., Pérez-García S., Rodríguez-Viejo J. (2010). Temperature dependent thermal conductivity of polycrystalline ZnO films. J. Appl. Phys..

[24] Zehentbauer F.M., Moretto C., Stephen R., Thevar T., Gilchrist J.R., Pokrajac D., Richard K.L., Kiefer J. (2014). Fluorescence spectroscopy of Rhodamine 6G: Concentration and solvent effects. Spectrochim. Acta A Mol. Biomol. Spectrosc..

[25] Ali M., Moghaddasi J., Ahmed S. (1991). Temperature effects in Rhodamine B dyes and improvement in CW dye laser performance. Laser Chem..

[26] Hamzaoui N., Boukhachem A., Ghamnia M., Fauquet C. (2017). Investigation of some physical properties of ZnO nanofilms synthesized by micro-droplet technique. Results Phys..

[27] Ghifari N., Chahboun A., El Abed A. One-Step Synthesis of Highly Monodisperse ZnO Core-Shell Microspheres in Microfluidic Devices. Proceedings of the 2019 21st International Conference on Transparent Optical Networks (ICTON).

[28] Duffy D.C., McDonald J.C., Schueller O.J., Whitesides G.M. (1998). Rapid prototyping of microfluidic systems in poly (dimethylsiloxane). Anal. Chem..

[29] Hayat Z., El Abed A.I. (2018). High-throughput optofluidic acquisition of microdroplets in microfluidic systems. Micromachines.

[30] Taylor G. (1961). Deposition of a viscous fluid on the wall of a tube. J. Fluid Mech..

[31] Baroud C.N., Gallaire F., Dangla R. (2010). Dynamics of microfluidic droplets. Lab Chip.

[32] Chu T.X., Salsac A.V., Barthès-Biesel D., Griscom L., Edwards-Lévy F., Leclerc E. (2013). Fabrication and in situ characterization of microcapsules in a microfluidic system. Microfluid. Nanofluidics.

[33] Hu X.Q., Salsac A.V., Barthès-Biesel D. (2012). Flow of a spherical capsule in a pore with circular or square cross-section. J. Fluid Mech..

[34] Liu Z., Wen X., Wu X., Gao Y., Chen H., Zhu J., Chu P. (2009). Intrinsic dipole-field-driven mesoscale crystallization of core- shell ZnO mesocrystal microspheres. J. Am. Chem. Soc..

[35] Tampo H., Fons P., Yamada A., Kim K.K., Shibata H., Matsubara K., Yoshikawa H., Kanie H., Niki S. (2006). Determination of crystallographic polarity of ZnO bulk crystals and epilayers. Phys. Status Solidi C.

[36] Dai S., Park H.S. (2013). Surface effects on the piezoelectricity of ZnO nanowires. J. Mech. Phys. Solids.

[37] Xiang H., Yang J., Hou J., Zhu Q. (2006). Piezoelectricity in ZnO nanowires: A first-principles study. Appl. Phys. Lett..

[38] Yufei Z., Zhiyou G., Xiaoqi G., Dongxing C., Yunxiao D., Hongtao Z. (2010). First-principles of wurtzite ZnO (0001) and (0001) surface structures. J. Semicond..

